# Hydrophobic Mismatch Controls the Mode of Membrane-Mediated Interactions of Transmembrane Peptides

**DOI:** 10.3390/membranes12010089

**Published:** 2022-01-13

**Authors:** Oleg V. Kondrashov, Peter I. Kuzmin, Sergey A. Akimov

**Affiliations:** Frumkin Institute of Physical Chemistry and Electrochemistry, Russian Academy of Sciences, 31/4 Leninskiy Prospekt, 119071 Moscow, Russia; copper1956@mail.ru

**Keywords:** lipid membrane, theory of elasticity, transmembrane domain, liquid-ordered domain, membrane-mediated interactions

## Abstract

Various cellular processes require the concerted cooperative action of proteins. The possibility for such synchronization implies the occurrence of specific long-range interactions between the involved protein participants. Bilayer lipid membranes can mediate protein–protein interactions via relatively long-range elastic deformations induced by the incorporated proteins. We considered the interactions between transmembrane peptides mediated by elastic deformations using the framework of the theory of elasticity of lipid membranes. An effective peptide shape was assumed to be cylindrical, hourglass-like, or barrel-like. The interaction potentials were obtained for membranes of different thicknesses and elastic rigidities. Cylindrically shaped peptides manifest almost neutral average interactions—they attract each other at short distances and repel at large ones, independently of membrane thickness or rigidity. The hourglass-like peptides repel each other in thin bilayers and strongly attract each other in thicker bilayers. On the contrary, the barrel-like peptides repel each other in thick bilayers and attract each other in thinner membranes. These results potentially provide possible mechanisms of control for the mode of protein–protein interactions in membrane domains with different bilayer thicknesses.

## 1. Introduction

Various vital cellular functions are catalyzed by membrane proteins. The most complicated processes require the concerted cooperative action of several protein molecules, synchronized both spatially and temporally [1,2,3,4]. The possibility of such synchronization implies the occurrence of specific long-range interactions of the involved proteins. Membrane proteins usually disturb the membrane, thereby inducing elastic deformations, the characteristic lengths of which are about several nanometers [5,6,7]. Such lengths substantially exceed the typical length of electrostatic interactions—about 1 nm under physiological conditions. Thus, bilayer lipid membranes provide the medium and fundamental physical forces for the long-range interactions of membrane proteins.

Plasma membranes of mammalian cells are laterally heterogeneous [8,9,10]. Lipids and proteins are thought to be segregated into specific domains. Domains enriched in sphingomyelin and cholesterol are called rafts [11]. Cellular rafts have a conservative lipid and protein composition that allows membrane proteins to be classified as raft or non-raft [12,13]. In purely lipidic model membranes, which composition resembles the lipid composition of the outer leaflets of plasma membranes, micron-sized domains can be formed during the course of phase separation after being induced by a temperature drop [5,14,15]. The domains are considered a model of cellular rafts. Below, we use the terms “domains” and “rafts” synonymously. It has been established that lipids in model domains are in the liquid-ordered state as opposed to the liquid-disordered surrounding membrane. The transition from a disordered to an ordered state leads to an increase in the thickness of the lipid bilayer by about 0.5–1 nm [16,17]. This difference in thickness allows transmembrane proteins to choose the membrane domain of optimal thickness of the lipid bilayer, depending on the length of the protein’s transmembrane domain (TMD). A possible mismatch between the TMD length and the hydrophobic thickness of the lipid bilayer could lead to membrane deformations aimed at eliminating the contact of polar (water or lipid heads) and hydrophobic (lipid tails or TMD) media. The deformations arising in the TMD vicinity lead to the growth of system free energy. The tendency of the system to minimize elastic energy represents the driving force for the lateral sorting of proteins between membrane domains of different thicknesses; it is referred to as hydrophobic matching [18,19]. This type of sorting is very sensitive to even tiny differences in the bilayer thickness and the length of the TMD. In [20], variation in the TMD length by a single amino acid residue is shown to essentially affect the sorting.

Vital cellular processes require the simultaneous participation of several raft-associated membrane proteins. Among these processes are signal transduction [21,22,23], polarized sorting [24], and the creation of immunological synapses [25]. Some of these processes are stimulus-triggered. Importantly, before stimulation, the participating proteins did not interact with each other, and it is the stimulus that initiated their tight interaction. This may imply that rafts provided spatial separation of the participants before the stimulation. After the process is triggered-on, the fusion of rafts may gather all participants in a small restricted region of the membrane. Various vital processes require fusion of domains; they proceed incorrectly upon disturbance of the fusion. For example, in Smith-Lemli-Opitz syndrome [26], the metabolic precursor of cholesterol, 7-dehydrocholesterol, is accumulated in membranes [27]; higher levels of 7-dehydrocholesterol lead to an increase in the energy barrier of the ordered domain fusion [5] and affect the proper transduction of cellular signals [28,29].

However, the fusion of domains comprising all proteins involved is not sufficient for the process to proceed reliably, because even after appearing in the same raft, the proteins do not necessarily all come into close contact. In addition, the mechanisms for promoting the fusion of specific domains in response to the stimulus are not fully understood. If cellular rafts have similar lipid compositions, while they comprise very different proteins, it is not clear how to selectively induce the fusion of rafts comprising specific proteins involved in a particular process. It might be suggested that each type of protein creates a domain of unique lipid composition, which depends on subtle details of the protein structure. In other words, cellular rafts may not be the result of global phase separation in the plasma membrane but rather of the local phase separation induced by membrane proteins in their immediate vicinity, e.g., by the wetting mechanism [30,31,32,33]. However, this leads to the idea that the membrane must have a huge number of domain types, approximately equal to the number of membrane protein types. These domains should not merge spontaneously but should reliably fuse after the arrival of the stimulus; besides, the stimulus should only affect certain types of domains. However, in typical model lipid membranes formed from the “canonical raft mixture” (e.g., dioleoylphosphatidylcholine: sphingomyelin: cholesterol 1:1:1), the ordered domains fuse following nearly every collision [5,34]. The substitution of cholesterol by 7-dehydrocholesterol completely prevents the fusion of domains, but this leads to the impairment of cellular signaling, yielding heavy pathologies at the level of the organism [5,26,28,29]. Additionally, if there is a large number of different lipid domain types surrounding the membrane proteins, the lipid compositions of the domains cannot differ strongly. The stimulus acting on the lipid subsystem is unlikely able to select a particular lipid composition to modify it in a way that favors domain fusion.

A simpler way to regulate the aggregation of membrane proteins follows the suggestion that the stimulus should act on the protein and alter the protein conformation. Generally, protein TMD induces elastic deformation of the membrane. If the proteins are significantly separated, their induced deformations are independent, and their energy is additive. Upon the mutual approach of proteins, the deformations start to overlap, leading to effective membrane-mediated lateral interactions [35,36,37,38,39,40]. Depending on the shape of the TMD and the elastic parameters of the membrane, these interactions may be attractive, repulsive, or combined, e.g., long-range repulsion and short-range attraction [35,36,37,38,39,40]. It is assumed that, in the non-activated state, the structure of the TMD-induced elastic deformations corresponds to protein–protein repulsion. Upon ligand-triggered activation, the TMD conformation may alter in a way that favors protein–protein attraction and aggregation. Such a hypothetical mechanism provides the required high selectivity, as the stimulus acts immediately on the target proteins and does not affect the others. The mechanism does not imply the existence of numerous types of lipid domains; the interacting proteins may reside in either the ordered or the disordered membrane phase. The membrane-mediated interactions are relatively long-range [35,36,37,38,39,40], which allows the activated proteins to find each other effectively on the membrane.

**Figure 1:**
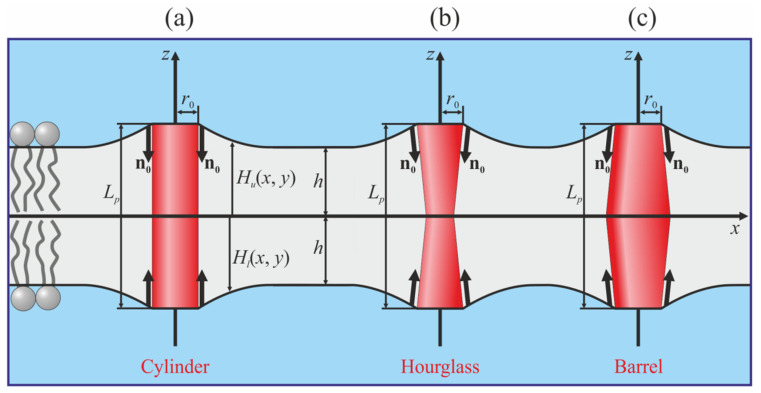
Schematic representation of the cylindrical (**a**), hourglass-like (**b**) and barrel-like (**c**) TMDs incorporated into the lipid membrane. Parameterization of elastic deformations of the membrane and boundary conditions at the TMDs are illustrated.

In the present work, we considered the interactions between transmembrane proteins mediated by elastic deformations of the membrane. Effective TMD shapes were assumed to be cylindrical, hourglass-like, and barrel-like (Figure 1).

We analyzed the interaction energy profiles in membranes with different bilayer thicknesses and elastic moduli. The characteristic shapes of the profiles appeared to be weakly dependent on the values of the elastic moduli but strongly dependent on the bilayer thickness. The interaction energy profiles of cylindrically shaped TMDs manifested long-range repulsion and short-range attraction. The hourglass-like TMDs were found to repel each other in thin bilayers and strongly attract each other in thicker bilayers. On the contrary, the barrel-like TMDs were shown to repel each other in thick bilayers and attract each other in thinner membranes. Hourglass-like and barrel-like TMDs interacted with each other similarly to cylindrical TMDs. These results potentially provide a possible mechanism for the selective tuning of protein–protein interactions in membrane domains with different bilayer thicknesses.

## 2. Materials and Methods

In order to calculate the energy of elastic deformations induced by TMDs, we utilized the theory of elasticity of lipid membranes originally developed by Hamm and Kozlov [41] and generalized it to include additional deformational modes [35,37,42]. In this theory, each monolayer of the membrane is considered a continuous elastic medium. The average orientation of anisotropic lipid molecules is described by unit vectors called directors, **n**. The field of directors is set at the neutral surface of the lipid monolayer, described by its unit normals, **N**. The neutral surface is located in the junction regions of polar heads with hydrophobic tails of lipids [43]. The monolayer may be subjected to some lateral tension, *σ*, acting along its neutral surface. We introduced a Cartesian coordinate system *Oxyz* where the *z*-axis is assumed to be perpendicular to the plane of the undeformed membrane, and the plane *Oxy* coincides with the monolayer interface surface of the undeformed membrane. The shape of the neutral surface is characterized by the *z*-coordinates of its points, described by the function *H*(*x*, *y*) (Figure 1). We accounted for the following elastic deformations of the monolayer: (I) splay or bending, determined by div(**n**) along the neutral surface; (II) tilt, determined by the deviation of **n** from **N**, described in the first order by the tilt-vector **t** = **n** − **N**; (III) lateral stretching, determined by the relative deviation of the area of the neutral surface, *α*; (IV) Gaussian splay; and (V) twist, determined by **rot**(**n**). The deformations were deemed small, and we worked under the framework of the linear theory of elasticity. The elastic energy of the deformed monolayer can be written as [35,37,42]

(1) $$\begin{array}{l} W=\int dS\{\frac{B}{2}{\left[\mathrm{div}\left(n\right)+J_{0}\right]}^{2}-\frac{B}{2}J_{0}^{2}+\frac{K_{t}}{2}t^{2}+\frac{\sigma }{2}{\left[grad\left(H\right)\right]}^{2}+\\ +\frac{K_{a}}{2}\alpha^{2}+K_{G}K+\frac{K_{rot}}{2}{\left[rot\left(n\right)\right]}^{2}\}, \end{array}$$

where *B*, *K_t_*, *K_a_*, *K_G_*, and *K_rot_* are the moduli of splay, tilt, lateral stretching, Gaussian splay and twist, respectively; *J*_0_ is the spontaneous curvature of the lipid monolayer, characterizing the preferable shape of the monolayer in the absence of external forces and torques; $K=\frac{\partial n_{x}}{\partial x}\frac{\partial n_{y}}{\partial y}-\frac{\partial n_{x}}{\partial y}\frac{\partial n_{y}}{\partial x}$ is the Gaussian splay (*n_x_* and *n_y_* are the corresponding projections of the director); and the integration is performed over the neutral surface of the monolayer. We assumed that the hydrophobic part of the lipid monolayer is locally volumetrically incompressible, as the bulk modulus of the membrane is very high, ~10^10^ J/m^3^ ≈ 3 × 10^3^ *k_B_T*/nm^3^ [44], where *k_B_T* ≈ 4.14 × 10^–21^ J. The condition of volumetric incompressibility imposes a restriction on the deformations [35,36,37,41,42]:

(2) $$\begin{array}{l} H_{u}-M=h-\frac{h^{2}}{2}\mathrm{div}\left(n_{u}\right)-h\alpha_{u},\\ M-H_{l}=h-\frac{h^{2}}{2}\mathrm{div}\left(n_{l}\right)-h\alpha_{l}, \end{array}$$

where *M* = *M*(*x*, *y*) is the shape of the monolayer interface and *h* is the hydrophobic thickness of the lipid monolayer. Here, and below the index, “*u*” corresponds to the upper monolayer located in the half-space *z* > 0, and the index “*l*” corresponds to the lower monolayer, which is located in the half-space *z* < 0. In the linear approximation, the vectors of the unit normal to the neutral surfaces can be written as $N_{u,l}={\left(\pm \partial H_{u,l}/\partial x,\pm \partial H_{u,l}/\partial y,\mp 1\right)}^{T}$ (the upper signs correspond to the upper and the lower signs to the lower monolayer). By expressing *α_u_*_,*l*_ from the incompressibility conditions shown in Equation (2), substituting *α_u_*_,*l*_ along with normals into the elastic energy functional, Equation (1), and accounting for two monolayers, one can obtain the elastic energy functional of the bilayer:

(3) $$\begin{array}{l} W=\int dS_{u}\{\frac{B}{2}{\left[\mathrm{div}\left(n_{u}\right)+J_{0}\right]}^{2}-\frac{B}{2}J_{0}^{2}+\frac{K_{t}}{2}{\left[n_{u}-grad\left(H_{u}\right)\right]}^{2}+\frac{\sigma }{2}{\left[grad\;\left(H_{u}\right)\right]}^{2}+\\ +\frac{K_{a}}{2h^{2}}{\left[h-\frac{h^{2}}{2}\mathrm{div}\left(n_{u}\right)+M-H_{u}\right]}^{2}+K_{G}K_{u}+\frac{K_{rot}}{2}{\left[rot\left(n_{u}\right)\right]}^{2}\}+\\ +\int dS_{l}\{\frac{B}{2}{\left[\mathrm{div}\left(n_{l}\right)+J_{0}\right]}^{2}-\frac{B}{2}J_{0}^{2}+\frac{K_{t}}{2}{\left[n_{l}+grad\left(H_{l}\right)\right]}^{2}+\frac{\sigma }{2}{\left[grad\;\left(H_{l}\right)\right]}^{2}+\\ +\frac{K_{a}}{2h^{2}}{\left[h-\frac{h^{2}}{2}\mathrm{div}\left(n_{l}\right)-M+H_{l}\right]}^{2}+K_{G}K_{l}+\frac{K_{rot}}{2}{\left[rot\left(n_{l}\right)\right]}^{2}\}. \end{array}$$

Far from the TMDs, we set the boundary conditions of an unperturbed membrane:

(4) $$n_{u,l}\left(\infty \right)=\left(0,0,\mp 1\right),\;H_{u}\left(\infty \right)=h,\;H_{l}\left(\infty \right)=-h.$$

At the boundary of the vertical cylindrical TMD, the following specific boundary conditions were set (Figure 1a):

(5) $$n_{u}(r=r_{0})=0,\;n_{l}(r=r_{0})=0,\;H_{u}(r=r_{0})-H_{l}(r=r_{0})=L_{p},$$

where *r* is the radial coordinate; *r*_0_ and *L_p_* are the radius and length of the TMD, respectively. Note, that vertical cylindrical TMD does not deform the membrane if *L_p_* = 2*h*. We denote the normal and tangential components of the boundary director as *n_n_* and *n_t_*, respectively. Then, the conditions at the boundary of the vertical hourglass-like or barrel-like TMD can be written as

(6) $$n_{n}^{u,l}\left(r=r_{0}\right)=n_{n0},\;n_{t}^{u,l}\left(r=r_{0}\right)=0,\;H_{u}\left(r=r_{0}\right)-H_{l}\left(r=r_{0}\right)=L_{p};$$

*n_n_*_0_ < 0 corresponds to the hourglass-like TMD (Figure 1b), while *n_n_*_0_ > 0 corresponds to the barrel-like TMD (Figure 1c). The boundary conditions of Equation (6) should be imposed at the boundaries of both TMDs. All three considered shapes of the TMD are mirror symmetric with respect to the monolayer interface. Consequently, the induced deformations should also possess the same symmetry, and, thus, we imposed the condition *M*(*x*, *y*) ≡ 0.

From the energy functional Equation (3), it follows that the energy contribution from the monolayer spontaneous curvature *J*_0_ can be transformed as

(7) $$W_{spont}=\int dS\left\{BJ_{0}\mathrm{div}\left(n\right)\right\}=-BJ_{0}\oint \left(n\cdot a\right)d\Gamma ,$$

where the last integration is performed over the TMD boundary contour Γ at the monolayer neutral surface; **a** is the outer unit normal vector to the contour. Thus, it follows that, for cylindrically shaped TMDs, the elastic energy is independent of the monolayer spontaneous curvature, as in this case **n** ⊥ **a** at the boundary contour, and thus, (**n·a**) ≡ 0. For hourglass-like or barrel-like shapes of TMDs, the corresponding contribution is a non-zero constant that is proportional to the monolayer spontaneous curvature. As we were interested in obtaining the interaction energy profiles, we further neglected this *J*_0_-related constant contribution to the elastic energy, as it only shifts the energy profiles without altering their shapes.

The elastic energy functional Equation (3) supplemented by the boundary conditions Equations (4)–(6) imposed on each TMD cannot be minimized analytically, as the symmetry of the system is too low. We minimized it numerically for a fixed, discreet set of distances between two TMDs, utilizing the finite element method with an adaptive mesh, similar to the methods used in works [35,37,42,45]. Briefly, we divided the plane *Oxy* into elementary triangles. Inside each triangle, the deformations were approximated by linear polynomials on coordinates, i.e., the deformations were replaced by their linear interpolants. We integrated the energy surface density in Equation (3) over each elementary triangle and obtained the total elastic energy as the sum over all triangles. To get the numerical value of the total elastic energy, the resulting function was minimized with respect to the values of deformations at mesh nodes, except for those set by the boundary conditions in Equations (4)–(6). The boundary conditions at infinity (Equation (4)) were actually set at a rectangle, the sides of which were at least 25 nm from TMDs. This distance substantially exceeds the typical decay length of membrane deformations, which is several nanometers [6,7,35]. To account for the inhomogeneous elastic energy density, we used inhomogeneous meshes: the surface density of nodes increased in the vicinity of TMDs. The intersections of TMDs with the neutral surfaces of monolayers were represented by a piecewise linear approximation. The neutral surfaces of lipid monolayers around the TMDs were subdivided into five regions, characterized by different levels of mesh fineness. Each region was specified by the inequality *r_i_*_−1_ ≤ *d* ≤ *_ri_*, where *d* is the distance to the boundary of the TMD; *r_i_*_−1_, *r_i_* are constants defining the inner and outer boundaries of the regions, respectively, for *i* = 1, …, 5. The numerical values of *r_i_* were *r*_0_ = 0; *r*_1_ = 1 nm; *r*_2_ = 1.5 nm; *r*_3_ = 4 nm; *r*_4_ = 11 nm; *r*_5_ = ∞. We restricted the maximum area of an elementary triangle of the computational mesh by 0.5*γ* (in nm^2^), and divided the regions defined above into elementary triangles of the area not exceeding *γθ_i_*, where *θ*_1_ = 0.01, *θ*_2_ = 0.02, *θ*_3_ = 0.04, *θ*_4_ = 0.05, *θ*_5_ = 0.5. This algorithm allowed the numerical value of the total elastic energy to be obtained for each value of *γ* set manually. We used the value *γ* = 1.5. Recently, we explicitly checked that the effect of the mesh fineness, *γ*, was insignificant for the membrane with incorporated TMD [45]. It was shown that the elastic energy calculated on the mesh described above differed from the value obtained from extrapolation to a zero mesh size by less than 1.5%. Thus, in the present work, we used computational mesh *γ* = 1.5 without extrapolation to an infinitely fine mesh.

In order to illustrate the results of calculations graphically, we utilized the following elastic parameter values: TMD length *L_p_* = 3.6 nm; TMD radius *r*_0_ = 0.65 nm. We considered three values for the normal component of the director at the TMD boundary: *n_n_*_0_ = 0 (cylindrical TMD), *n_n_*_0_ = +0.1 (barrel-like TMD), and *n_n_*_0_ = −0.1 (hourglass-like TMD) (Figure 1). To analyze the dependence of the TMD interaction on the elastic properties of the membrane, we used two sets of elastic moduli, approximately corresponding to liquid-disordered (index “*d*”) and liquid-ordered (index “*o*”) phases: splay moduli *B_d_* = 10 *k_B_T*, *B_o_* = 50 *k_B_T* [46,47]; lateral stretching moduli $K_{a}^{d}$ = 133 mN/m, $K_{a}^{o}$ = 665 mN/m [46,48]; tilt moduli $K_{t}^{d}$ = $K_{t}^{o}$ = 40 mN/m [41]; the moduli of the Gaussian splay were expressed via the splay moduli as *K_G_* = −*B*/2 [49] (although the Gaussian splay contribution is constant for the boundary conditions in Equations (4)–(6)); the twist moduli were estimated as *K_rot_* = *B*/2 [35,37,39,45]. We carried out the calculations for three hydrophobic lipid monolayer thicknesses: medium *h*_0_ = *L_p_*/2 = 1.8 nm; thin *h*_1_ = 1.5 nm; and thick *h*_2_ = 2.1 nm.

In our model, the hydrophobic thickness is the key parameter characterizing the lipid monolayer. The smallest of the considered hydrophobic thicknesses *h*_1_ = 1.5 nm corresponds approximately to the hydrophobic thickness of the monolayer formed from dioleoylphosphatidylcholine [46]. The intermediate thickness *h*_0_ = 1.8 nm may be attributed to the lipid monolayer of the liquid-ordered phase enriched by sphingomyelin and cholesterol [16,17]. Both the intermediate thickness and the largest hydrophobic thickness, *h*_2_ = 2.1 nm, may be achieved in membranes formed from lipids with long chains occurring in brain lipid extracts. However, for our model, only the relative differences in the TMD length and the bilayer thickness were substantial (see Equations (2), (5) and (6)). Formally, one can fix the bilayer thickness and consider the membrane-mediated interactions of TMDs of different lengths.

## 3. Results

**Figure 2:**
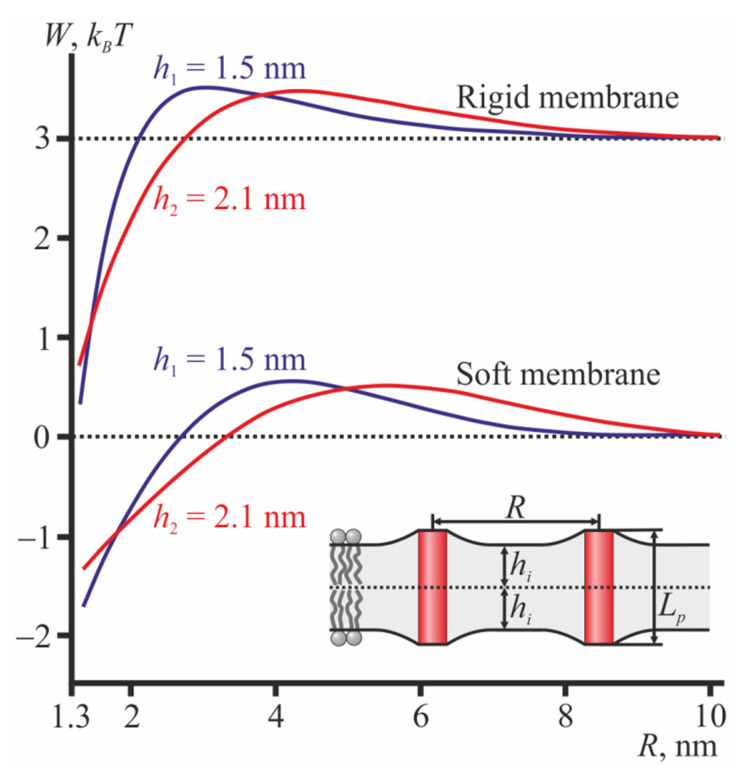
The interaction potentials of two cylindrical TMDs. Top set of the curves—in the ordered (rigid) membrane; bottom set of the curves—in the disordered (soft) membrane. Blue curves—*h*_1_ = 1.5 nm; red curves—*h*_2_ = 2.1 nm. For the medium monolayer thickness, *h*_0_ = 1.8 nm, no elastic deformations were induced by the cylindrical TMDs; thus, the elastic energy was equal to zero (horizontal dotted lines). The top set of curves is shifted upwards by the constant 3 *k_B_T* to distinguish it from the bottom set of curves. The inset illustrates two cylindrical TMDs in the thin bilayer; *L_p_* = 2*h*_0_ = 3.6 nm.

The dependence of the energy of membrane elastic deformations induced by two cylindrical TMDs on the distance *R* between their centers (axes of rotational symmetry) is presented in Figure 2. The energy values at *R* → ∞ were subtracted from the dependences to represent the interaction potentials; the dependences calculated for the rigid membrane were shifted by the constant value to give a better appearance. From Figure 2, it can be seen that when *L_p_* ≠ 2*h*, the interaction of the cylindrically shaped TMDs is generally long-range repulsion and short-range attraction. When 2*h* = *L_p_*, no elastic deformations are induced, and the interaction energy is strictly zero.

In order to evaluate the type of average interaction (attraction or repulsion) under low TMD concentrations, one should calculate the Mayer’s first cluster integral [50,51,52]: $\beta_{1,AB}=\int 2\pi R\left(e^{-\frac{w_{AB}\left(R\right)}{k_{B}T}}-1\right)dR$, where *R* is the distance between the centers of two TMDs (A and B) and *w_AB_* is the interaction potential of the TMDs. Positive values of *β*_1,*AB*_ correspond to an average attraction, while negative values correspond to average repulsion of the TMDs. However, from the curves of the interaction potentials (Figure 2), it can generally be concluded that, on average, cylindrical TMDs interact rather weakly as short-range attraction is compensated for (at least, partially) by long-range repulsion. The membrane rigidity influences the positions of the potential minima and maxima as well as their relative amplitudes but does not affect the qualitative features of the potentials (i.e., long-range repulsion and short-range attraction).

**Figure 3:**
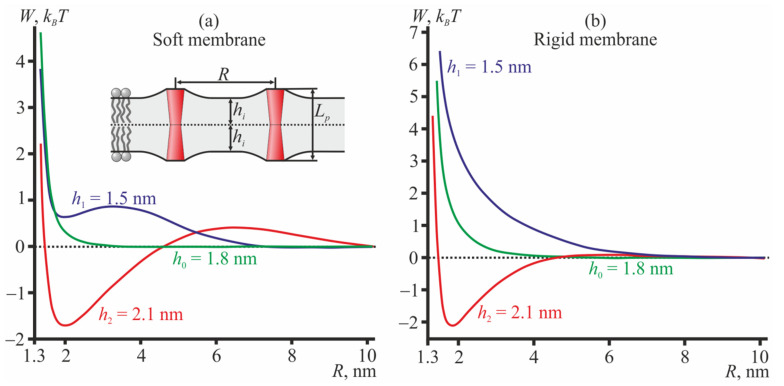
The interaction potentials of two hourglass-like TMDs (**a**) in the disordered (soft) membrane; (**b**) in the ordered (rigid) membrane. The monolayer hydrophobic thicknesses are shown as follows: blue curves—*h*_1_ = 1.5 nm; green curves—*h*_0_ = 1.8 nm; red curves—*h*_2_ = 2.1 nm. The inset in panel (**a**) illustrates two hourglass-like TMDs in the thin bilayer; *L_p_* = 2*h*_0_ = 3.6 nm.

For TMDs with the hourglass-like shape, the interaction potentials changes notably (Figure 3). Such TMDs repel each other in the thin bilayer (*h*_1_ = 1.5 nm) and medium thickness bilayer (*h*_0_ = 1.8 nm) and attract each other in the thick bilayer (*h*_2_ = 2.1 nm) in both soft (Figure 3a) and rigid (Figure 3b) membranes. The membrane rigidity influences the positions of the minima and maxima potentials as well as their relative amplitudes, but does not influence the qualitative regime (i.e., attraction or repulsion) of the TMD interaction. Note that the monolayer thicknesses should be considered in a relative sense. Actually, the interaction potentials shown in Figure 3 can be interpreted as indicating that in a bilayer of some fixed thickness 2*h*, hourglass-like TMDs of length *L_p_* ≥ 2*h* should repel each other while TMDs of length *L_p_* < 2*h* should mutually attract each other. The absolute values of the monolayer thickness influence the characteristic lengths of deformations, i.e., they affect the positions of the energy minima and maxima. The qualitative regime of TMD interactions (i.e., attraction or repulsion) depends on the differences in the TMD length and the bilayer thickness.

**Figure 4:**
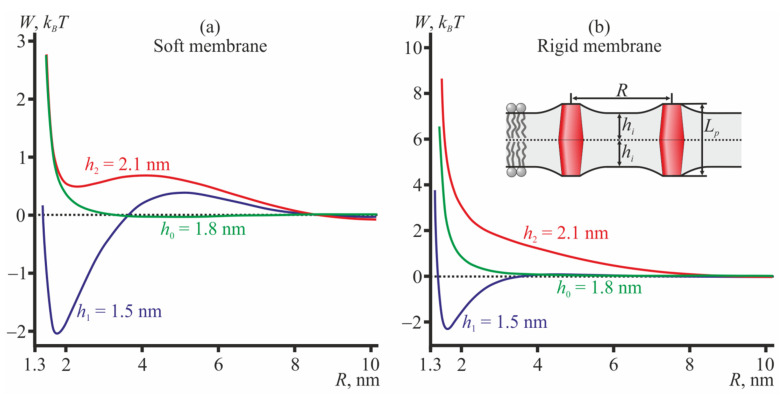
The interaction potentials of two barrel-like TMDs (**a**) in the disordered (soft) membrane; (**b**) in the ordered (rigid) membrane. The monolayer hydrophobic thicknesses are shown as follows: blue curves—*h*_1_ = 1.5 nm; green curves—*h*_0_ = 1.8 nm; red curves—*h*_2_ = 2.1 nm. The inset in panel (**b**) illustrates two barrel-like TMDs in the thin bilayer; *L_p_* = 2*h*_0_ = 3.6 nm.

For TMDs with a barrel-like shape, the dependence of the interaction potentials on the monolayer thickness is the opposite (Figure 4): barrel-like TMDs attract each other in the thin bilayer (*h*_1_ = 1.5 nm) and repel each other in the thick bilayer (*h*_2_ = 2.1 nm) and in the medium thickness bilayer (*h*_0_ = 1.8 nm) in both soft (Figure 4a) and rigid (Figure 4b) membranes.

These results may also be interpreted as showing that in a bilayer of some fixed thickness 2*h*, barrel-like TMDs of length *L_p_* ≤ 2*h* should repel each other, while TMDs of length *L_p_* > 2*h* should mutually attract each other.

Note, that the depth of the energy minimum of attractive curves (red curves in Figure 3; blue curves in Figure 4) is almost independent of the membrane rigidity, in all cases being about 2 *k_B_T* if counted from the energy at *R* → ∞. However, the values of the elastic moduli affect the height of the energy maxima of these curves at *R* ≈ 5–7 nm; the maxima heights vary in the range 0.05–0.5 *k_B_T*.

**Figure 5:**
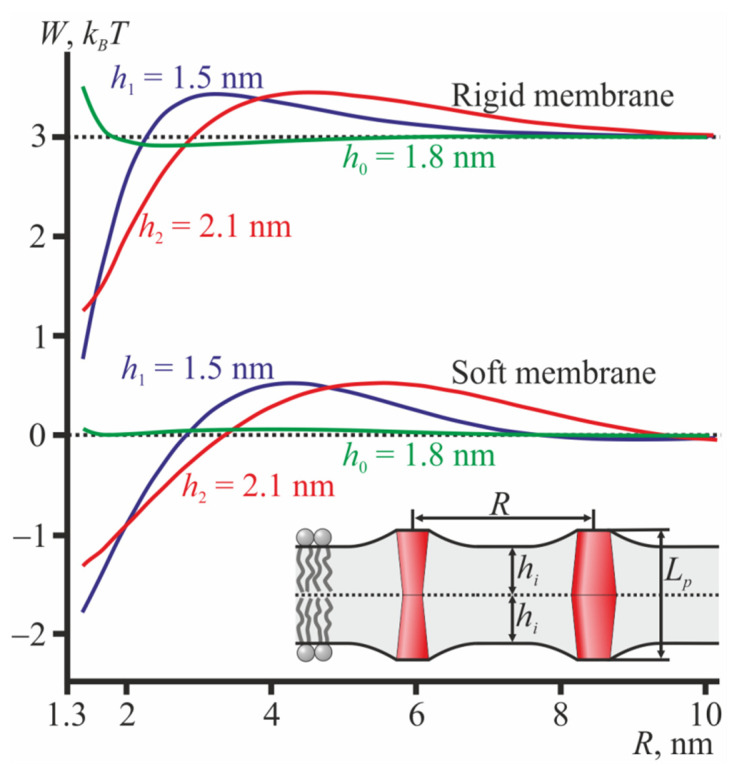
The interaction potentials of hourglass-like and barrel-like TMDs. Top set of curves—in the ordered (rigid) membrane; bottom set of curves—in the disordered (soft) membrane. Blue curves—*h*_1_ = 1.5 nm; green curves—*h*_0_ = 1.8 nm; red curves—*h*_2_ = 2.1 nm. The top set of curves is shifted upwards by the constant 3 *k_B_T* to distinguish it from the bottom set of curves. The inset illustrates hourglass-like and barrel-like TMDs in the thin bilayer; *L_p_* = 2*h*_0_ = 3.6 nm.

When two interacting TMDs have different shapes, i.e., one is barrel-like while the other is hourglass-like, their interaction potentials are similar to those obtained with two interacting cylindrical TMDs (Figure 5).

From Figure 5, it can be seen that when *L_p_* ≠ 2*h*, the interaction of the hourglass-like and barrel-like shaped TMDs is generally long-range repulsion and short-range attraction. When 2*h* = *L_p_* the interaction is very weak and exhibits some degree of repulsion at short distances in rigid membranes.

## 4. Discussion

In the present work, we analyzed the interaction of cylindrical, hourglass-like, or barrel-like transmembrane inclusions mediated by elastic deformations of the membrane. Different membrane thicknesses (relative to the length of the TMD) and different elastic rigidities were considered. For interacting hourglass-like and barrel-like TMDs, as well as for cylindrical TMDs, the interaction manifested as long-range repulsion and short-range attraction; on average, the interaction appeared almost neutral. The hourglass-like TMDs demonstrated repulsive interaction in bilayers of small (2*h*_1_ = 3 nm) and medium (2*h*_0_ = 3.6 nm) thicknesses, while they were mutually attracted to each other in thick (2*h*_2_ = 4.2 nm) bilayers. On the contrary, the barrel-like TMDs were attracted to each other in thin (2*h*_1_ = 3 nm) bilayers and experienced repulsive interactions in bilayers of medium (2*h*_0_ = 3.6 nm) and large (2*h*_2_ = 4.2 nm) thicknesses (Figure 4). The modes of interaction weakly depended on the membrane elastic rigidity for all three types of transmembrane inclusions (Figure 2, Figure 3, Figure 4 and Figure 5). Of note, the interaction potentials of hourglass-like and barrel-like TMDs exhibited strong repulsion when there was a small distance between the TMDs (Figure 3 and Figure 4). However, these results should be considered with care, as when the distance between the peptides is smaller than the lateral size of a lipid molecule (~0.7 nm), the application of the continuum theory of elasticity becomes incorrect.

**Figure 6:**
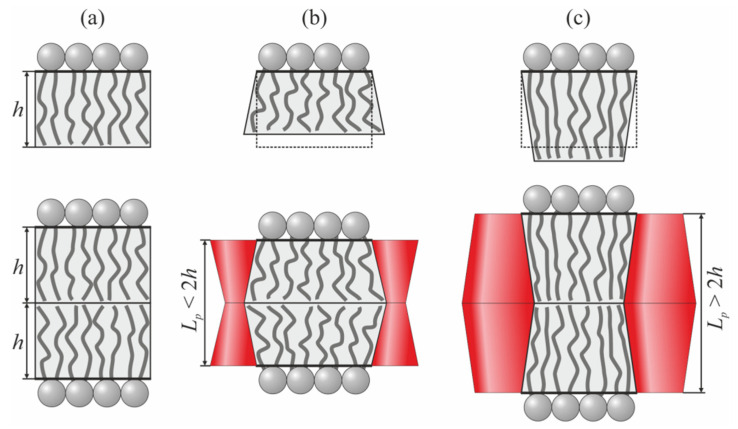
The local volumetric incompressibility allows the monolayer element thickness to be matched to half of the TMD length via transformation of the rectangular element (**a**) to a trapezium-like shape with either: (**b**) a larger bottom base (decreased thickness) or (**c**) a smaller bottom base (increased thickness). The former trapezium fits in better between the hourglass-like TMDs, the length of which is less than the bilayer thickness (**b**), while the latter trapezium fits in better between the barrel-like TMDs, the length of which is greater than the bilayer thickness (**c**).

The opposing modes of interaction for hourglass-like and barrel-like TMDs in thin and thick membranes are the fundamental consequence of the local volumetric incompressibility (Equation (2)) of lipid membranes. The lateral stretching modulus of the lipid monolayer is large compared to the characteristic energy density of splay deformation. Indeed, the lateral stretching modulus is *K_a_* ~ 133 mN/m ≈ 32 *k_B_T*/nm^2^, while the splay modulus divided by the square of the monolayer thickness is *B*/*h*^2^ ~ 10/1.5^2^ *k_B_T*/nm^2^ ≈ 4.5 *k_B_T*/nm^2^. This means that the membrane is hardly laterally stretchable. Thus, the least energy demanding way for the rectangular monolayer element (Figure 6a) to adjust its thickness is to transform into a trapezium at the expense of splay deformation, keeping the area of the neutral surface constant. Transformation to a trapezium with a large bottom base leads to a decreased thickness (Figure 6b), while transformation to a trapezium with a smaller bottom base yields a larger monolayer thickness (Figure 6c). This formally follows on from the conditions of local volumetric incompressibility, Equation (2). In our case of symmetric deformations with respect to the monolayer interface, the interface remains flat, i.e., *M*(*x*, *y*) ≡ 0. If, for simplicity, we prohibit the lateral stretching of the monolayer neutral surface (i.e., *α_u_* = *α_l_* = 0), the conditions of Equation (2) can be rewritten for the upper monolayer as

(8) $$H_{u}-h=-\frac{h^{2}}{2}\mathrm{div}\left(n_{u}\right).$$

For the upper monolayer, the trapezium with a larger bottom base corresponds to positive div(**n***_u_*) and, consequently, to a decreased monolayer thickness, (*H_u_* − *h*) < 0 (Figure 6b). The trapezium with a smaller bottom base corresponds to negative values of div(**n***_u_*), yielding an increased monolayer thickness, (*H_u_* − *h*) > 0 (Figure 6c).

A similar consideration can be made for the bottom monolayer. The trapezium with the larger bottom base fits in better between the hourglass-like TMDs, which leads to the attraction of such TMDs in thicker bilayers (Figure 6b), as it is the element of the thicker bilayer that has to transform into a trapezium to adjust (decrease) its thickness to fit the length of the TMD. On the contrary, the trapezium with the smaller bottom base fits in better between the barrel-like TMDs, which leads to attraction of such TMDs in thinner bilayers, as it is the element of the thinner bilayer that has to transform into a trapezium to adjust (increase) its thickness to fit the length of the TMD (Figure 6c).

Practically, the cylindrical TMDs can be related to peptides with charged anchoring residues, like KALP [53,54]. Hourglass-like TMDs model peptides with bulky tryptophan anchoring residues, like WALP [53,54] or dimer of gramicidin A (gA) [55,56]. In [40], the interaction of two gramicidin dimers was analyzed by means of the molecular dynamics (MD) in membranes formed from dimiristoylphosphatidylcholine, dipalmitoylphosphatidylcholine, and distearoylphosphatidylcholine. The thickness of all three bilayer was found to be greater than the length of the gramicidin dimer [40]. The interaction potentials determined from MD qualitatively coincide with the interaction potential obtained within our continuum elastic model for hourglass-like TMDs in thick bilayers (red curves in Figure 3). From experiments with tandem channels formed by two lateral dimers of gramicidin located in opposing monolayers of the membrane, it is known that the channel lifetime increases steeply as two dimers approach each other. In experiments with covalent dimers (two monomers located in opposing membrane monolayers linked at the membrane midplane) of minigramicidin (gA analogue cut by 4 amino acids from the N-terminus), it has been shown that the lifetime of such short covalent dimers increases by about 40 times when the lifetime is determined in the ensemble as compared to single channel experiments [57]. Considering our results, these data may be interpreted as showing that the covalent dimers of minigramicidin have an effective hourglass-like shape, and being incorporated into a relatively thick bilayer, they strongly attract each other, yielding greater channel lifetimes.

As barrel-like TMDs, we consider peptides with bulky hydrophobic amino acid residues (e.g., isoleucine) in the region of the monolayer interface, peptides subjected to posttranslational modification (i.e., stearoylation, palmitoylation, miristoylation), and/or those comprising so-called CRAC-motifs thought to bind to cholesterol [58,59,60]. Both posttranslational modification and cholesterol binding should increase the relative volume of the hydrophobic part of the peptide, thus yielding an effective barrel-like shape. The transmembrane domain of influenza fusion protein, hemagglutinin, has both the CRAC-motif and the palmitoyl residue attached [61,62,63] that allow its shape to be modeled as barrel-like. The length of the hemagglutinin TMD is about 27 amino acid residues [61,64,65], which corresponds to the length of the α-helix—about 4.05 nm [66]. The membrane of the influenza virion is thought to be in the liquid-ordered state, and thus, its typical hydrophobic thickness should be about 3.6 nm [67,68], i.e., less than the length of the hemagglutinin TMD. For barrel-like TMDs with a relatively thin bilayer, our model predicts mutual attraction (blue curves in Figure 4). Hemagglutinin-induced membrane fusion is a cooperative process involving 3 to 9 hemagglutinin trimmers aggregated into a so-called fusion rosette [69]. We speculate that the attractive mode of interaction for barrel-like TMDs in a thin bilayer might be responsible for the formation of the fusion rosette, even in cases of low concentrations of hemagglutinin trimmers in the membrane.

We consider the radius of the TMD to be equal to *r*_0_ = 0.65 nm, which approximately corresponds to the radius of the α-helix. However, for TMDs of other sizes, the interaction energy should scale approximately with the TMD radius, analogously to the interaction energy scaling obtained for short amphipathic peptides [42].

The membrane-mediated interactions of two cylindrical inclusions were considered in detail in [39]. The authors utilized a slightly different elastic model and explicitly considered the consequences of the structural restrictions of lipid chains in the vicinity of the solid membrane inclusion. Nevertheless, their obtained interaction energy profiles are very similar to the potentials calculated within our model and presented in Figure 2.

The interaction potentials of conical membrane inclusions were derived analytically in [38]. It was shown that the equally oriented conical inclusions spanning the bilayer always repel each other, which is in agreement with our results for hourglass-like TMDs in bilayers with medium (2*h*_0_ = 3.6 nm) and small (2*h*_1_ = 3 nm) thicknesses (Figure 3, green and blue curves) and barrel-like TMDs with medium (2*h*_0_ = 3.6 nm) and large (2*h*_2_ = 4.2 nm) thickness bilayers (Figure 4, green and red curves). However, in [38], the membrane was considered to be an infinitely thin film; under such a framework, the attractive mode of interaction cannot be principally anticipated, as the attractive regime originates from the local volumetric incompressibility of the membrane.

Of note, our findings and predictions do not depend on the actual phase state (L_o_ or L_d_) of the membrane. The model uses the monolayer thickness, spontaneous curvature, and moduli of elasticity. Generally, the L_o_ phase is characterized by a somewhat larger monolayer thickness and more rigid membranes. However, increased thickness can be achieved in the L_d_ phase through the use of lipids with longer hydrocarbon chains, and their rigidity can be independently tuned by the degree of the chain unsaturation. For this reason, we considered all combinations of thickness (thick, medium, and thin monolayers) and elastic rigidity (soft and rigid membranes), including a thick monolayer with low rigidity and a thin monolayer with high elastic rigidity, in order to make the consideration independent of the validity of the hypothesis of L_o_/L_d_ phase coexistence in biological membranes. Moreover, for our model, only the relative differences in the TMD length and bilayer thickness were substantial. Formally, one can fix the bilayer thickness and consider the membrane-mediated interactions of TMDs of different lengths, yielding predictions similar to those illustrated in Figure 2, Figure 3, Figure 4 and Figure 5. Such a consideration is completely independent of the particular phase state of the membrane and, thus, does not rely on the raft hypothesis.

Cellular plasma membranes are highly occupied by transmembrane and peripheral proteins. Thus, it seems that, in biological membranes, relatively long-range membrane-mediated interactions should involve multiple proteins. However, as we showed recently [70], the typical energy of membrane-mediated interactions involving peripheral membrane inclusions is about an order of magnitude smaller than the energy of interactions of transmembrane inclusions. If transmembrane and peripheral proteins have similar surface concentrations, the contribution of peripheral proteins to the total energy of membrane-mediated interactions should be small. Nevertheless, multiple protein–protein interactions may become substantial when the surface density of TMDs is high. The multiple interactions can be accounted for on the basis of Mayer cluster expansion, which allows the partition function of the system to be calculated as a series of interacting particle concentrations, the coefficients of which can be expressed via the pairwise interaction potentials of the particles [50,51,52]. These pairwise potentials were calculated in our work and are presented in Figure 2, Figure 3, Figure 4 and Figure 5. In this sense, our work qualitatively describes the interaction of transmembrane peptides in the limit of low concentration and provides a basis for the calculation of the partition function of the system for proteins with increasing surface density.

Cellular plasma membranes contain about 100 different types of lipid, that have chains with different lengths and degrees of unsaturation, and are characterized by different degrees of elastic rigidity and spontaneous curvature [71]. Generally, hydrophobic mismatch and the effects of non-cylindrical protein shapes can be compensated for by local enrichment of the TMD vicinity by specific lipids of appropriate length and spontaneous curvature. In this case, no deformation of the membrane should arise, thus leading to the absence of membrane-mediated interactions of TMDs. Intuitively, if compensation for the effect of the non-cylindrical shape of a TMD can take place alternatively at the expense of bending deformation or local redistribution of lipids, one should compare the corresponding moduli, i.e., the bending modulus of monolayer (~10 *k_B_T*) and the typical energy of thermal motion (~1 *k_B_T*). Such a comparison leads to the incorrect conclusion that the lateral redistribution of membrane components costs an order of magnitude less energy than the deformation of the membrane patch in the TMD vicinity, and thus, elastic deformations should not arise. Actually, one should not compare the moduli, but rather, the typical energies or energy surface densities. For example, in the approximation of an ideal solution, the local enrichment of the lipid component with an average concentration (mole fraction) of *x*_10_ = 0.3 to the local concentration in the TMD vicinity of *x*_1_ = 1.0 should cost about (*x*_1_*k_B_T*ln[*x*_1_/*x*_10_] + (1 − *x*_1_)*k_B_T*ln[(1 − *x*_1_)/(1 − *x*_10_)])/*a*_0_ ≈ 1.7 *k_B_T*/nm^2^ (here *a*_0_ ≈ 0.7 nm^2^ is the area per lipid molecule at the monolayer surface) [72]. The corresponding bending energy density can be estimated as *BJ_s_*^2^/2 ≈ 10 *k_B_T*·(1/3 nm^–1^)^2^/2 ≈ 0.2 *k_B_T*/nm^2^ (here *J_s_* is the spontaneous curvature of the lipid component, roughly estimated as |*J_s_*|~1/3 nm^–1^, which is typical for cholesterol and dioleoylphsphatidylethanolamine) [73]. This means that it is much easier to deform the membrane rather than laterally redistribute the membrane components. Practically, both deformation and lateral redistribution should contribute to the compensation for the effects of the non-cylindrical shape of the TMD. However, as the energy density of deformation is less than that of the lateral redistribution of membrane components, deformations should definitely arise in the TMD vicinity, thus leading to membrane-mediated long-range lateral interactions of TMDs. The lateral redistribution of the components can only slightly dampen the deformations, yielding decreases in their characteristic lengths.

Plasma membranes are asymmetric with respect to the lipid compositions of their constituent monolayers. The outer monolayer is enriched with electrically neutral phosphocholine lipids with saturated chains, while the inner monolayer is enriched with negatively charged unsaturated lipids, phosphoethanolamines, etc. [71]. Generally, the outer and inner monolayers of plasma membranes should possess different monolayer thicknesses, spontaneous curvatures, and elastic rigidities. Qualitatively, the calculated TMD interaction potentials are weakly dependent on the membrane rigidity (Figure 2, Figure 3, Figure 4 and Figure 5). The spontaneous curvature of the lipid monolayer yields the only constant contribution to the elastic energy (Equation (7)) and does not influence the interaction potential. The results of our calculations depended strongly on the hydrophobic thickness of the lipid monolayers (Figure 2, Figure 3, Figure 4 and Figure 5). However, as we considered the transmembrane peptides, we set the boundary conditions for the total bilayer (rather than monolayer) thickness at the TMD boundary (see Equations (5) and (6)). Thus, only the total bilayer hydrophobic thickness is substantial, while the exact thicknesses of each monolayer of the membrane are not important.

Cholesterol is a major lipid component of plasma membranes of mammalian cells [71]. Generally, cholesterol increases the membrane thickness and elastic rigidity, although the effect depends on the degree of lipid chain saturation/unsaturation. Additionally, cholesterol induces highly negative spontaneous curvature in lipid monolayers [73]. Spontaneous curvature gives a constant contribution to the elastic energy of the membrane (Equation (7)), which is independent of the distance between the TMDs and, thus, does not influence the interaction potentials. A cholesterol-induced increase in the elastic rigidity should slightly alter the interaction energy profiles (Figure 2, Figure 3, Figure 4 and Figure 5). However, the interaction potentials depend strongly on the bilayer thickness (Figure 2, Figure 3, Figure 4 and Figure 5). The change in the bilayer thickness upon cholesterol addition should depend on the initial amount of cholesterol present in the membrane. If, initially, the soft bilayer contains no cholesterol, while the rigidity of the rigid bilayer is provided by the high cholesterol content, the addition of cholesterol should increase the thickness of the soft bilayer to a larger extent than for the rigid bilayer. This would alter the interaction potentials of hourglass-like and barrel-like TMDs (Figure 3 and Figure 4). However, for cylindrical TMDs, the interaction potentials in the thin and thick bilayers are qualitatively similar (Figure 2). Thus, if cholesterol addition does not yield an exact match for the bilayer thickness and the length of the cylindrical TMD, the addition should result in no qualitative change to the interaction potentials in the soft and rigid bilayers.
